# Supplementary material for: Cytokine profiling of samples positive for Chlamydia trachomatis and Human papillomavirus
Source: PLoS One. 2023 Mar 10;18(3):e0279390. doi: 10.1371/journal.pone.0279390 (PMC10004564; doi:10.1371/journal.pone.0279390)
Supplement: S1 Table — (PDF) [file pone.0279390.s001.pdf]

S1 Table – Cytokines concentrations from coinfected individuals HPV/Chlamydia.

ID	Citokines (pg/ml)													
	IL-17		IFN-γ		TNF		IL-10		IL-6		IL-4		IL-2	
	Blood	Cervix	Blood	Cervix	Blood	Cervix	Blood	Cervix	Blood	Cervix	Blood	Cervix	Blood	Cervix
89	35.00	0.00	2.25	0.00	0.50	1.24	1.19	0.00	1.36	296.42	0.00	0.00	0.14	0.00
176	13.58	0.00	0.00	0.00	0.11	0.00	0.96	0.00	1.24	21.79	0.00	0.00	0.00	0.00
268	19.30	13.58	2.95	0.00	2.23	0.11	2.25	0.00	3.23	2.38	2.33	0.00	2.51	0.00
