# Supplementary material for: Cytokine profiling of samples positive for Chlamydia trachomatis and Human papillomavirus
Source: PLoS One. 2023 Mar 10;18(3):e0279390. doi: 10.1371/journal.pone.0279390 (PMC10004564; doi:10.1371/journal.pone.0279390)
Supplement: Attachment — Submitted filename: In this manuscript 3-21-22.docx [file pone.0279390.s002.docx]

In this manuscript, the authors described the quantification of the profile of Th1/Th2/Th17 cytokine in exfoliated cervix cells (ECC) and in peripheral blood (PB) of patients positive for Chlamydia trachomatis (Ct) DNA, patients positive for Papillomavirus DNA, and healthy patients. The results replicate other previous published works with some new data to the field. The paper has provided useful information on immune response to important STI pathogens, Ct and HPV. However, a reduced number of samples, the lack of details in patient and sample detections, and the limited interpretations on the results make it hard to draw the strong conclusions. In addition, some reference were displaced or improper. Some comments to improve the quality of this study are as below.

Major Concerns：

Methods:

1. Patients: What is the age range of the individuals?
2. What is the rationale behind the PCR testing HPV together with the human β-globin gene?
3. Lines 166 and 538: a wrong reference (23) for Ct primers and detection.

Results:

1. If could be nice to include the cytokine levels from the individuals that had co-infection of Ct and HPV.
2. Lines 267-268: the tittle was unclear here. Perhaps it should be “comparison of cytokine levels in samples positive for *C. trachomatis* DNA with those in HPV DNA-positive samples”.
3. Table 2: column 4 “*C. tracomatis* negative” should be healthy control or no infections?
4. If could be interesting to compare whether the cytokine levels with the individuals that had HPV DNA were different from the healthy controls.
5. Figures 1 and 2 were blurred.

Minor：
1. Lines 77-78: “the elementary body (EB) that is extracellular, metabolically inactive…” this is not true. Recent evidence suggest that EB has low metabolic ability.  Omsland A, Sixt BS, Horn M, Hackstadt T.FEMS Microbiol Rev. 2014 Jul;38(4):779-801. doi: 10.1111/1574-6976.12059. Epub 2014 Feb 24.

2. Line 92: use a reference with women study is better.
