# Supplementary material for: Cytokine profiling of samples positive for Chlamydia trachomatis and Human papillomavirus
Source: PLoS One. 2023 Mar 10;18(3):e0279390. doi: 10.1371/journal.pone.0279390 (PMC10004564; doi:10.1371/journal.pone.0279390)
Supplement: Attachment — Submitted filename: Response to Reviewers.docx [file pone.0279390.s004.docx]

# **Response to Review’s Comments**

(Manuscript Number: PONE-D-22-04538

"**Cytokine profiling of samples positive for Chlamydia trachomatis and Human papillomavirus**" (Original Article)

Dear Editor,

We appreciated the suggestion from reviewer about our manuscript. Please find below the answer to his specific comments/suggestions/queries.

***Response to Reviewer #2 Comment***

1. The authors have made the best attempts to address comments raised by reviewers and have satisfactorily provided responses. However, given the nature of the study design and the limitations therein as indicated by the authors in the revision, it is advised to include a paragraph in the discussion section highlighting study design related caveats and the endeavor to undertake future/ ongoing studies to address them. This will serve as a good start point for other research groups investigation similar pathogens or study designs.

**Response: “This study is not intended to be definitive or conclusive on the subject. Still, it contributes to understanding more about the interaction between the immune response and these two microorganisms, if the cervical or systemic microenvironment can somehow influence viral persistence and if the presence of C. Trachomatis can modulate this environment. The work aimed to consider the cytokines produced by the different groups with samples collected from the cervix and blood. However, studies by our group associated immunophenotyping with cytokine dosage to characterize better the patterns obtained. One of these studies is already published [50].” We added this information in the discussion section (Lines 421-429).**
